# Patient with Dravet syndrome: A case report

**DOI:** 10.1002/ccr3.5840

**Published:** 2022-05-05

**Authors:** Rukesh Yadav, Sangam Shah, Bibek Bhandari, Kundan Marasini, Prince Mandal, Hritik Murarka, Anuj Kumar Pandey, Basanta Sharma Paudel

**Affiliations:** ^1^ Maharajgunj Medical Campus Institute of Medicine Tribhuvan University Maharajgunj Nepal; ^2^ Department of Internal Medicine Maharajgunj Medical Campus Institute of Medicine Tribhuvan University Maharajgunj Nepal; ^3^ Department of Radiology Maharajgunj Medical Campus Institute of Medicine Tribhuvan University Maharajgunj Nepal; ^4^ 92959 Tribhuvan University Teaching Hospital Maharajgunj Nepal

**Keywords:** dravet, SCN1A, syndrome

## Abstract

Dravet syndrome is rare genetic epilepsy syndrome and epileptic encephalopathy. The patient initially has normal developmental profile with plateau or regression that begins after seizure onset. We report a case of two‐year‐old child diagnosed as dravet syndrome with moderate cerebral atrophy and ventricular dilatation as rare MRI finding.

## Abstract 2

Cerebral atrophy and ventricular dilatation can be present in MRI of brain in patient with DS. The patients with DS are undertreated and do not get the standard treatment of DS because of misdiagnosis.

## 1 INTRODUCTION

Dravet syndrome (DS) is an epileptic encephalopathy that typically manifests as febrile seizures in early childhood. First described by Charlotte Dravet in 1978, DS is a genetic epilepsy syndrome characterized by neurodevelopmental problems due to refractory epilepsy. DS is a rare disorder affecting 1 in 15,700 to 1 in 40,000 live births with equal preponderance in males and females.^1^, ^2^, ^3^ The main features include refractory epilepsy that is characterized by multiple different types of seizure (including tonic‐clonic, hemiclonic, generalized clonic, focal impaired awareness, myoclonic, and absence seizures), neurodevelopmental delay and neurologic disability that begin after onset of seizure, and cognitive and motor system dysfunction persisting into adulthood. Majority of the patients have mutations in the voltage‐gated sodium channel alpha‐1 subunit (SCN1A) gene.^4^, ^5^ We report a case of two‐year‐old child diagnosed as dravet syndrome with moderate cerebral atrophy and ventricular dilatation as rare MRI finding. We also highlight the core clinical features, course, and management of DS in context of Nepal.

## 2 CASE PRESENTATION

A 2‐year‐old male patient presented to our center with chief complaints of abnormal body movement for 3 days and on/off fever for 2 days. He also had urinary incontinence. On examination, he was ill‐looking, unresponsive, and not well oriented to time, place, and person. He had up rolled eyes with vacant stare, open mouth, drooling of saliva, stiffening, and abnormal movement of right upper and lower limb. On general physical examination, he had pallor. He had no rashes, petechiae, purpura or bruises, icterus, lymphadenopathy, cyanosis, clubbing, edema, and dehydration.

Physical examination revealed heart rate of 142 beats per minutes, respiratory rate of 28 breaths per minute, blood pressure of 100/70 mm of Hg, temperature of 100.2^0^F, and oxygen saturation of 99% on oxygen facemask maintained at the rate of 4 L/min. His airway was patent, and there was no sign of respiratory distress or evidence of trauma. He was admitted to pediatric intensive care unit (PICU) because of status epilepticus and was sedated with midazolam immediately.

On further examination, he had global developmental delay. He had no head control with head being turned to one side, he did not grasp finger or reached for objects, and he recognized his mother and cooed. He had learning difficulty; he was not able to speak words as he should be able to as per his age. He had delayed motor development; he was not able to perform motor activities appropriate for his age. Anthropometry revealed head circumference of 46.5 cm, body weight of 10 kg, and height of 83 cm, of which head circumference and height were lower than expected approximate values.

Ophthalmological examinations were normal with no features of disk edema. His cranial nerves were intact. There were no signs of meningeal irritation. Motor examination revealed increased tone in upper limb, which was relatively high on extension than on flexion. However, increased tone in lower limb was noted that was high on flexion than on extension. Babinski's extensor response was present bilaterally. Other systemic examinations were normal.

Laboratory examination revealed hemoglobin 9.9 g%, packed cell volume 32.9%, total red blood cell count 490,000 cells/mm^3^, total leukocyte count 3800 cells/mm^3^ with neutrophils 48%, lymphocytes 42%, monocytes 9%, eosinophil 1%, basophil 0%, platelets 256,000 cells/mm^3^, urea 5.8 mmol/l, 0.5 creatinine mg/dl, Na^+^142 mEq/L, K^+^ 3.8 mEq/L, total protein 70 g/L, and albumin 49 g/L. His random blood glucose and calcium level were 112 mg/dl and 9.6 mg/dl, respectively. C‐reactive protein (CRP) latex was negative. There was no growth of microorganisms in blood after 72 h. Cerebrospinal fluid (CSF) analysis showed total leucocyte count of 8 cells/mm^3^, protein and glucose levels were within the normal range. Urine analysis had cystine (3+) crystal/hpf.

**FIGURE 1:**
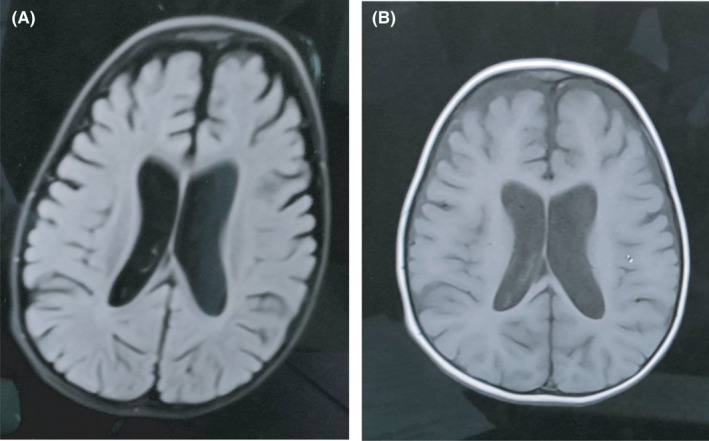
(A) FLAIR (B) T1 weighted showing proportionate dilatation of the ventricles and moderate cerebral atrophy

Interictal electroencephalography (EEG) was done during wakefulness that showed no significant findings. Magnetic resonance imaging (MRI) of the brain showed prominent extra‐axial CSF spaces with proportionate dilatation of the ventricles consistent with global cerebral atrophy (Figure 1). Genetic analysis showed SCN1A gene mutation.

The boy is the second child of a non‐consanguineous parent following the first girl child who is healthy. The mother had hydatidiform mole. She had 3 successive spontaneous abortions after the birth of first child. She did not smoke, consumed alcohol, or used illicit or teratogenic drugs during the period of gestation. She had undergone all necessary examination and antenatal visits. He was born at term following emergency cesarean section due to fetal distress. He did not cry at birth. His birthweight and head circumference were normal at the time of birth. He was discharged after 4 days following evaluation and management in the neonatal intensive care unit (NICU). The mother and father were 26 and 38 years old, respectively, at the time of gestation. He has been fully immunized according to the expanded program on immunization (EPI) schedule of Nepal.

The patient had first episode of seizure at 2 months of age. He also had fever after vaccination. He used to have up to 24 episodes of seizure per day. He was on levetiracetam and on a regular follow‐up since then. His developmental milestones were normal till 18 months of age. Later, he lost his head control. He also used to have occasional stiffening of head toward right, abnormal flexion of all limbs, lip smacking, and chewing movement.

He was diagnosed with dravet syndrome. Following this, he was managed with ceftriaxone (250 mg/day), levetiracetam (125 mg/day), clobazam (0.625 mg/day), sodium valproate (50 mg/day), and paracetamol (125 mg per rectal).

## 3 DISCUSSION

In Nepal, DS was not reported previously because it is rare, difficult to diagnose, or is misdiagnosed as a febrile seizure or other epileptic syndromes, or because there is no follow‐up and genetic tests are not available.

The child had seizures since the age of two months (up to 24 episodes for 1 day), refractory to most of the antiseizure medications, began as alternating hemiclonic, generalized tonic‐clonic seizure later followed by absence seizure. The seizure used to last for more than 10 min. He had normal early developmental profile that began to regress after seizure onset. Thus, the patient was suspected to have DS and recommended to get genetic testing done.

DS can be mistaken for other form of seizure including febrile seizures. Febrile seizures, lennox‐gastaut syndrome (LGS), myoclonic‐astatic epilepsy, progressive myoclonus epilepsy (PME), severe infantile multifocal epilepsy (SIMFE), and PCDH19‐related epilepsy are the common differential diagnosis of DS. These differentials were ruled out based on history, clinical features, examination findings, and laboratory investigations. DS can be diagnosed on a clinical basis between the second year of life and the plateau phase.

According to the Dravet Syndrome Foundation^6^ and other medical bodies, the diagnostic criteria for this condition should include several of the following symptoms: (1) onset of seizures in the first year of life in an otherwise healthy infant. The average age of onset is 5.2 months; (2) initial seizures that are typically prolonged and are generalized or unilateral; (3) presence of other seizure types (i.e., myoclonic seizures, tonic‐clonic or hemiconvulsive seizures); (4) seizures associated with fever due to illness or vaccinations; (5) seizures induced by prolonged exposure to warm temperatures; (6) seizures in response to strong lighting, photosensitivity or certain visual patterns; (7) initially normal EEGs and later EEGs with slowing and severe generalized polyspikes; (8) normal initial development followed by slow development during the first few years of life; (9) some degree of hypotonia; (10) unstable and crouched gait and balance issues; (11) an MRI may be normal or show mild generalized atrophy and/or hippocampal sclerosis; (12) ankle pronation and flat feet and/or development of a crouched gait with age; (13) in older children and adults, persisting seizures, which may or may not be prolonged. Status epilepticus becomes less frequent with time and may not be apparent by young adulthood; (14) MRI may be normal or show mild generalized atrophy and/or hippocampal sclerosis; (15) two or more seizures with or without fever before 1 year of age; (16) two or more seizures lasting longer than 10 minutes; (17) failure to respond to first‐line antiepileptic drug therapy with continued seizures after 2 years of age. In our patient, several of the abovementioned features were present.

Infants with DS have normal physical and psychomotor development at the time of the first seizure which occurs usually between five and eight months of age while our patient developed seizure at the age of 2 months.^7^, ^8^, ^9^ Febrile tonic‐clonic seizure is the most common type of during first year of life. Uncommonly, some patients may have myoclonic and dyscognitive seizures. Seizures are often prolonged, sometimes leading to status epilepticus. Seizures are precipitated by fever/illness, immunization, and bathing in the first year of life.^9^ As the child grows, he/she will have multitude seizure types, and fever, emotional stress; flashes of light or overexertion are the seizure precipitants. The child with DS will subsequently develop hypotonia, ataxia, incoordination, and pyramidal signs, dysautonomia events, cognitive impairment, and behavioral disturbances like attention deficit, hyperactivity, or autistic traits.

Approximately 80% of patients with DS have mutation in SCN1A gene which is located on chromosome 2q24. Truncating mutations (40%), missense mutations (40%), nonsense mutations, and splice site changes are the most common genomic abnormalities in SCN1A gene. Other genes involved in remaining patients with DS are PCDH19, SCN1B, SCN2A, GABRA1, STXBP1, CDH2 and rarely HCN1, KCNA2, and GABRG2.^9^, ^10^, ^11^, ^12^, ^13^, ^14^, ^15^ Our patient had SCN1A gene mutation which is the commonest mutation seen in DS.

The EEG performed during early phases of the disease is normal. However, as the child grows, it may show slowing of background activity and poly‐spike or generalized spike wave pattern. MRI of the brain is normal at initial presentation but rarely it can demonstrate enlarged ventricles with cerebral atrophy as seen in our case.^16^

Our patient had classical presentation of DS with infantile onset (two months of age) of alternating hemiclonic, generalized tonic‐clonic seizure and status epilepticus which was later followed by absence seizures. He had normal early developmental profile that began to regress after seizure onset and exacerbation in the first year of life. Autonomic manifestations like urinary incontinence can occur in DS.

Despite the fact that antiseizure medications have limited efficacy and drug resistance, every effort should be made to minimize seizure triggers and control seizures and status epilepticus as much as possible. Stiripentol, valproate, benzodiazepine, and topiramate are among the drugs that can help reduce the frequency of seizures and the severity of the disease. Cannabidiol, fenfluramine, and bromides are among the newer drugs for the management of seizure in patients with DS.^17^, ^18^, ^19^ Besides medication, controlling infections and body temperature variations also showed to decrease the frequency of seizures and severity of the disease. Ketogenic diet and neuromodulation are viable options in selected patients, but still pharmacologic therapy remains the mainstay of treatment in patient with DS. Drugs acting on sodium channels like carbamazepine and its analogues, and phenytoin should be avoided as these drugs have potential to worsen the seizure in DS patients.^20^, ^21^ Vaccination should not be withheld in children with DS. It is worth noting that many of the patients are undertreated and do not get the standard DS treatment because of the misdiagnosis.

## 4 CONCLUSION

DS has overall poor outcome but considering seizure types, triggering factors, developmental profile, response to drugs and other management modalities, long‐term outcome and comorbidities are unique to each child, they should be treated accordingly. Hippocampal sclerosis, cerebellar, and cerebral atrophy, enlarged ventricles, focal cortical dysplasia, and increased white matter signal can rarely be reported in patient with DS. All family members (affected and unaffected) should undergo genetic mutation analysis, but the high cost of the genetic mutation analysis is the limiting factor for many of them in the low‐income countries like Nepal.

## AUTHOR CONTRIBUTIONS

RY and SS wrote the original manuscript, reviewed, and edited the manuscript. BB and KM reviewed and edited the original manuscript. RY, SS, BB, KM, PM, HM, AKP, and BSP reviewed the manuscript and were involved in the management of the case.

## CONFLICT OF INTEREST

None.

### ETHICAL APPROVAL

None.

### CONSENT

Written informed consent was obtained from the patient to publish this report in accordance with the journal's patient consent policy.

## Data Availability

All the required information is in the manuscript itself.
